# Awareness and preparedness for local anesthetic systemic toxicity among surgical clinicians: a multi-center cross-sectional survey study

**DOI:** 10.1186/s12871-026-04073-7

**Published:** 2026-07-03

**Authors:** Rasim Onur Karaoglu, Acelya Toprak Karaoglu, Sezen Kumas Solak, Mehmet Salih Sevdi, Serdar Demirgan

**Affiliations:** 1 Department of Anesthesiology and Reanimation, SBU Bağcılar Training and Research Hospital, Istanbul, Türkiye; 2 Department of Anesthesiology and Reanimation, Zeynep Kamil Hospital, Istanbul, Türkiye

**Keywords:** Local anesthetics, Drug toxicity, Regional anesthesia, Patient safety, Medical education, Clinical competence, Cross-sectional studies

## Abstract

**Background:**

Local Anesthetic Systemic Toxicity (LAST) is a rare but potentially fatal complication of regional anesthesia and local anesthetic infiltration. Despite established treatment protocols, the extent of clinician awareness and institutional preparedness across surgical specialties remains poorly characterized.

**Methods:**

This descriptive cross-sectional study enrolled 192 physicians from eight surgical specialties across five tertiary-level training and research hospitals in Türkiye (April–May 2025). A validated, structured questionnaire assessed LAST knowledge, clinical practices, and institutional readiness. Multivariable logistic regression identified independent predictors of correct lipid emulsion dosing knowledge, adjusting for specialty, seniority, and training status.

**Results:**

Only 41.1% of participants had received formal LAST training. Correct identification of the recommended lipid emulsion bolus dose (1.5 mL/kg) was reported by 34.4% overall, rising to 58.2% among trained participants versus 18.1% among untrained participants (*p* < 0.001). On multivariable analysis, formal training (OR 6.38; 95% CI 3.21–12.68), anesthesiology specialty (OR 3.14; 95% CI 1.67–5.91), and senior academic title (OR 2.07; 95% CI 1.08–3.96) were independent predictors of correct dose knowledge. Early symptom recognition was reported by 49.5%. Aspiration before injection was performed consistently by 53.1%, and ultrasound guidance by 38.5%. Lipid emulsion was readily available in only 29.2% of departments.

**Conclusion:**

Significant gaps exist in LAST knowledge, preventive practices, and institutional preparedness across surgical specialties. Targeted simulation-based education, mandatory availability of lipid emulsion, and standardized institutional protocols are warranted to improve patient safety.

**Supplementary Information:**

The online version contains supplementary material available at https://doi.org/10.1186/s12871-026-04073-7.

## Introduction

Local anesthetics (LAs) have become indispensable in contemporary surgical practice, providing targeted analgesia with minimal systemic effects in appropriately selected patients [1, 2]. Their widespread application, spanning peripheral nerve blocks, regional anesthesia, and minor surgical infiltration, has substantially improved patient comfort and enabled day-case surgery [3, 4]. However, this broad use carries a critical and frequently underestimated risk: Local Anesthetic Systemic Toxicity (LAST) [5, 6].

LAST is a rare yet potentially life-threatening complication arising from inadvertent intravascular injection, excessive dosing, or impaired drug metabolism [5–7]. Its clinical presentation spans a spectrum from subtle neurological prodrome, circumoral numbness, tinnitus, metallic taste, to severe cardiovascular collapse and refractory seizures [5, 6, 8]. Early recognition and prompt management, including airway protection, seizure control, and intravenous 20% lipid emulsion therapy, are essential determinants of outcome [6, 8, 9].

Despite clearly defined treatment guidelines and a growing evidence base, awareness of LAST remains suboptimal across multiple medical specialties [10–13]. Many clinicians are unfamiliar with safe dosage thresholds, early warning signs, and the logistics of initiating lipid rescue therapy [13]. This knowledge deficit may critically delay life-saving interventions [13, 14].

LAST is clinically relevant far beyond anesthesiology. General surgeons, orthopedic surgeons, obstetricians and gynecologists, otolaryngologists, urologists, and neurosurgeons all routinely administer local anesthetics, yet LAST education is not systematically integrated into most surgical training curricula [11, 13]. Preparation and verification practices also vary widely across institutions, introducing additional risk of dosing errors and delayed recognition [13, 14].

The present study aimed to assess awareness, knowledge, and institutional preparedness for LAST among physicians across eight surgical specialties at five tertiary-level hospitals in Türkiye, using a structured cross-sectional survey design. Specific objectives were: (1) to characterize the prevalence of formal LAST training and correct lipid emulsion dosing knowledge; (2) to identify specialty- and seniority-related differences in LAST awareness and preventive practices; (3) to evaluate institutional lipid emulsion availability; and (4) to determine independent predictors of LAST knowledge using multivariable logistic regression.

## Materials and methods

### Study design and setting

This was a descriptive cross-sectional survey study. Data were collected between April 15 and May 18, 2025, in surgical departments of five different tertiary-level training and research hospitals in Türkiye. The study protocol was approved by the Non-Interventional Clinical Research Ethics Committee of Medipol University (Decision No: E-10840098-202.3.02-2468; April 11, 2025) and was conducted in accordance with the Declaration of Helsinki. Written informed consent was obtained from all participants before enrollment.

### Participants and eligibility

Physicians actively working in surgical disciplines, anesthesiology, general surgery, orthopedics, obstetrics and gynecology, otolaryngology, neurosurgery, urology, and cardiovascular surgery, were invited to participate. These specialties were selected based on their routine and frequent use of local anesthetics in the participating institutions and their representation across the five study sites. Specialties such as plastic surgery, dermatology, and ophthalmology, which also administer local anesthetics, were not available at all five participating centers and were therefore excluded to maintain consistency across sites. This represents a limitation of the current sampling frame. Physicians were enrolled through consecutive voluntary sampling; all eligible clinicians present in the participating departments during the data collection period were invited to participate. Random sampling was not employed; therefore, volunteer bias cannot be excluded, and findings should be interpreted accordingly.

Inclusion criteria were: (1) active clinical practice in one of the eight specified surgical specialties at a participating hospital; and (2) willingness to complete the questionnaire in full. Exclusion criteria were: (1) practice in a non-surgical specialty; (2) medical intern status (not yet fully licensed); and (3) submission of an incomplete or invalid questionnaire. Questionnaire invalidity was defined a priori by both investigators prior to data collection based on the following criteria: more than 20% of items left unanswered; reporting no knowledge of local anesthetic dosing while simultaneously providing a specific numerical value for the lipid emulsion bolus or maintenance dose; reporting never performing aspiration before injection while simultaneously affirming always following preparation protocols; reporting knowledge of lipid emulsion location while simultaneously denying any knowledge of LAST management; or reporting a personal encounter with a LAST event while simultaneously denying any knowledge of early or late LAST symptoms. Flagged questionnaires were reviewed independently by both authors and excluded by consensus.

**Fig. 1:**
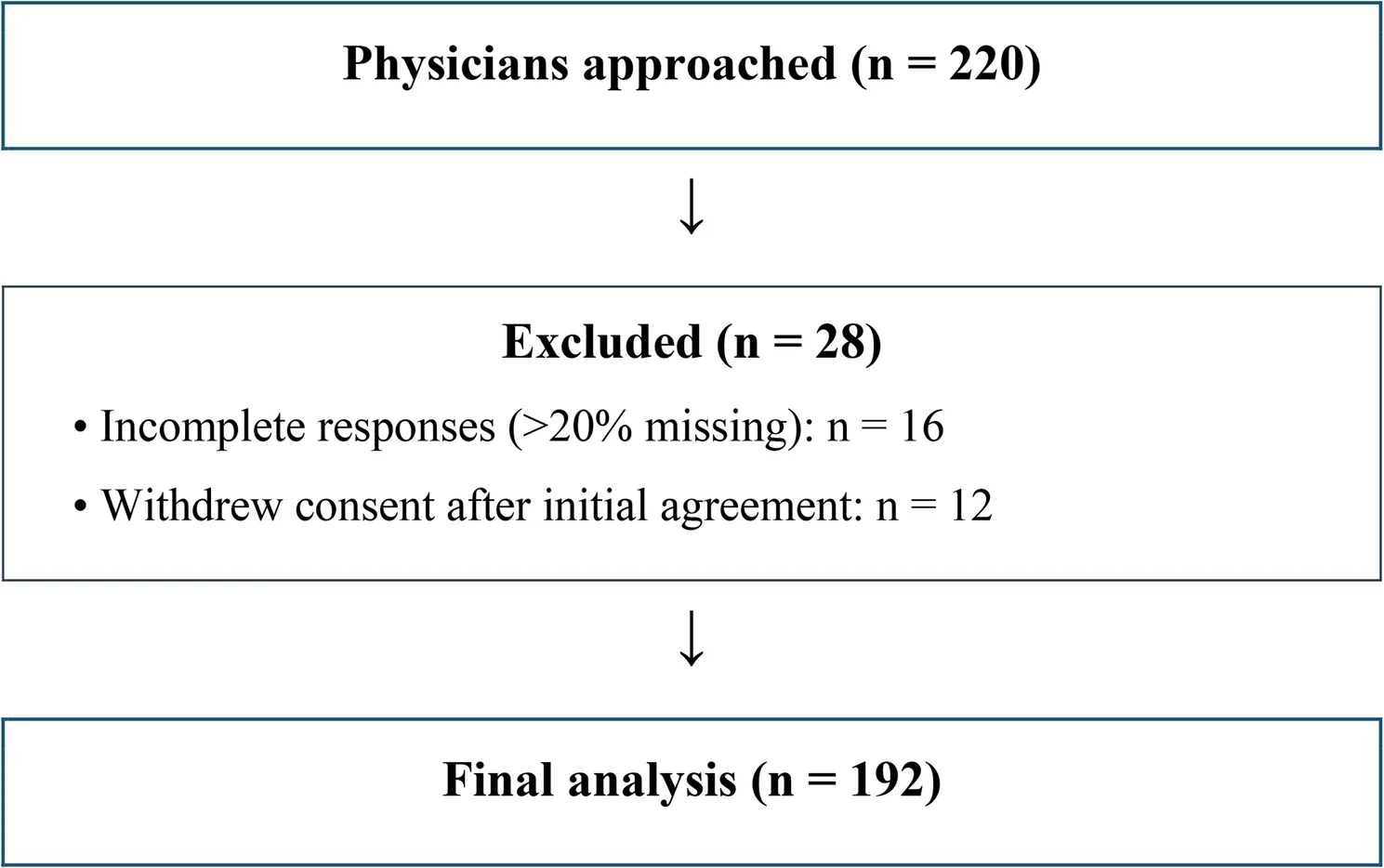
Participant flow diagram

As a formal a priori sample size calculation was not performed, the target number of approached physicians was determined pragmatically: approximately 220 physicians were approached based on the available physician workforce across the five participating institutions during the data collection period, aiming to achieve broad representational sampling across all eight specialties. This pragmatic approach is acknowledged as a limitation and is discussed further in the limitations section. After applying exclusion criteria, 192 valid questionnaires were retained for analysis (28 excluded: 16 due to incomplete responses, 12 due to withdrawal of consent after initial agreement). The participant flow is presented in Fig. 1.

### Study instrument

Data were collected using a structured, self-administered questionnaire developed by the investigators based on current ASRA guidelines [6] and relevant literature [5, 8, 10–14]. The full questionnaire is provided in Appendix A. An initial item pool was reviewed by two independent anesthesiologists for face validity and content relevance; items with disagreement were revised through consensus discussion. A pilot study with 10 eligible volunteers (not included in the final analysis) was conducted to assess clarity and internal consistency (Cronbach’s alpha = 0.74, indicating acceptable reliability).

The final questionnaire comprised eight sections: (1) demographic information (age, gender, specialty, academic title, years of experience); (2) formal LAST training (yes/no); (3) clinical practice patterns (agents used, frequency, routes); (4) preparation and verification protocols; (5) preventive strategies (aspiration before injection, ultrasound use); (6) recognition of early and late LAST symptoms; (7) management knowledge and lipid emulsion experience; and (8) institutional preparedness (lipid emulsion availability).

Gender was self-reported as male or female by participants. The primary outcome variable was correct identification of the recommended initial bolus dose of 20% lipid emulsion for LAST treatment (1.5 mL/kg), operationalized as a binary variable (correct vs. incorrect or unknown).

### Sample size

A formal a priori sample size calculation was not performed, as the primary goal was broad representational sampling across eight specialties and five institutions rather than hypothesis testing with a pre-specified effect size.

### Statistical analysis

All statistical analyses were performed using IBM SPSS Statistics, Version 26.0 (IBM Corp., Armonk, NY, USA). Continuous variables are reported as mean ± standard deviation (SD) or median (interquartile range, IQR) according to normality. Normality was assessed using the Kolmogorov-Smirnov and Shapiro-Wilk tests and Q-Q plot inspection. Categorical variables are expressed as frequencies and percentages.

Group comparisons used the chi-square test or Fisher’s exact test for categorical variables, and the independent-samples t-test or Mann-Whitney U test for continuous variables. A linear-by-linear chi-square trend test was applied for ordered categorical comparisons (e.g., academic seniority and preventive practices).

Multivariable binary logistic regression was performed with correct identification of the lipid emulsion bolus dose as the dependent variable. The following covariates were entered simultaneously based on a priori clinical reasoning: formal LAST training (yes/no), specialty (anesthesiology vs. other), academic title (senior [specialist/associate professor/professor] vs. resident), age (continuous), and gender. Results are presented as odds ratios (ORs) with 95% confidence intervals (CIs). Model calibration was assessed with the Hosmer-Lemeshow test. Subgroup analyses by specialty and academic title were pre-specified in the study protocol. A two-tailed *p* < 0.05 was considered statistically significant.

## Results

### Participant Characteristics

The primary outcome of this study was correct identification of the recommended initial bolus dose of 20% lipid emulsion for LAST treatment (1.5 mL/kg), operationalized as a binary variable (correct vs. incorrect or unknown). Of 220 physicians approached, 192 completed valid questionnaires (response rate: 87.3%). The median age of included participants was 31 years (IQR: 29–36); age was reported as median (IQR) given non-normal distribution (Shapiro-Wilk *p* = 0.03). Detailed demographic and educational characteristics are presented in Table 1. Residents constituted the largest academic subgroup (66.1%), and anesthesiology was the most represented specialty (40.1%). Formal LAST training had been received by 41.1% of participants.

**Table 1:** Demographic and educational characteristics (*n* = 192)

Variable	*n* (%)
Total participants	192 (100%)
Median age (years, IQR)	31 (29–36)
Gender	
Female	105 (54.7%)
Male	87 (45.3%)
Academic title	
Resident	127 (66.1%)
Specialist	47 (24.5%)
Associate Professor	15 (7.8%)
Professor	3 (1.6%)

Specialty	Trained, n (%)
Anesthesiology	77 (40.1%) 52 (67.5%)
General Surgery	26 (13.5%) 6 (23.1%)
Orthopedics	21 (10.9%) 5 (23.8%)
Obstetrics & Gynecology	20 (10.4%) 7 (35.0%)
Otolaryngology	16 (8.3%) 3 (18.8%)
Neurosurgery	13 (6.8%) 3 (23.1%)
Urology	11 (5.7%) 2 (18.2%)
Cardiovascular Surgery	8 (4.2%) 1 (12.5%)

### LAST knowledge and institutional readiness

LAST knowledge and institutional preparedness findings are summarized in Table 2. Correct identification of the recommended 20% lipid emulsion bolus dose (1.5 mL/kg) was achieved by only 66 participants (34.4%); 36.5% (*n* = 70) selected an incorrect dose and 29.2% (*n* = 56) reported not knowing the dose. Awareness of early LAST symptoms (perioral numbness, tinnitus, dizziness) was reported by 49.5% (*n* = 95). Consistent aspiration before injection was practiced by 53.1% (*n* = 102), and routine ultrasound guidance by 38.5% (*n* = 74). Lipid emulsion availability data by department are presented in Table 2.

**Table 2:** LAST knowledge and institutional readiness (*n* = 192)

Variable	*n* (%)
Recognition of early LAST symptoms	95 (49.5%)
Knowledge of lipid emulsion bolus dose (1.5 mL/kg)	
Correct (all participants)	66 (34.4%)
Incorrect	70 (36.5%)
Does not know	56 (29.2%)
Correct dose — by training status	
Trained participants (*n* = 79)	46 (58.2%)
Untrained participants (*n* = 113)	20 (18.1%)
Lipid emulsion availability in department	
Available	56 (29.2%)
Not available	74 (38.5%)
Unknown	62 (32.3%)
Preventive practices	
Performs aspiration before injection (always)	102 (53.1%)
Uses ultrasound guidance routinely	74 (38.5%)

### Lipid dose knowledge by specialty

Correct lipid dose knowledge varied substantially by specialty (Table 3). Anesthesiology participants had the highest correct response rate, while all other specialties demonstrated considerably lower accuracy. In non-anesthesiology specialties, more than 85% of participants either provided an incorrect dose or reported not knowing it.

**Table 3:** Correct knowledge of lipid emulsion dose by specialty

Specialty	*n*	Correct (%)	Incorrect (%)	Don’t Know (%)
Anesthesiology	77	39.0%	33.8%	27.2%
General Surgery	26	11.5%	42.3%	46.2%
Orthopedics	21	14.3%	38.1%	47.6%
Obstetrics & Gynecology	20	10.0%	45.0%	45.0%
Otolaryngology	16	6.3%	43.8%	50.0%
Neurosurgery	13	7.7%	46.2%	46.1%
Urology	11	9.1%	45.4%	45.5%
Cardiovascular Surgery	8	12.5%	50.0%	37.5%

### Effect of formal training on knowledge

Formal LAST training was significantly associated with correct lipid dose identification (Table 4). Among those who had received training, 58.2% (46/79) answered correctly, compared with only 18.1% (20/113) of untrained participants (χ²=34.7, df = 1, *p* < 0.001). A post-hoc power analysis confirmed that this comparison was adequately powered: with *n* = 79 trained and *n* = 113 untrained participants, the achieved power exceeded 99% (effect size h = 0.89, two-tailed alpha = 0.05). For the logistic regression model with five predictors and 192 observations, the events-per-variable ratio was approximately 9:1 (45 events), meeting minimum recommended thresholds.

**Table 4:** LAST training status vs. lipid dose knowledge

LAST Training	Correct Dose *n* (%)	Incorrect/Unknown *n* (%)	Total *n*
Yes	46 (58.2%)	33 (41.8%)	79
No	20 (18.1%)	93 (81.9%)	113
*p* value	< 0.001 (χ²=34.7, df = 1)		

### Preventive practices by academic title

Adherence to preventive practices showed a significant positive trend with increasing seniority (Table 5). Professors and associate professors reported the highest compliance with aspiration before injection (66.7% each) and ultrasound guidance (66.7% and 46.7%, respectively), while residents showed the lowest rates (aspiration 49.6%; ultrasound 35.4%). The trend across academic titles was statistically significant for both aspiration (*p* = 0.041) and ultrasound use (*p* = 0.038).

**Table 5:** Preventive practices by academic title

Academic Title	Aspiration Before Injection (%)	Ultrasound Guidance (%)
Resident (*n* = 127)	49.6%	35.4%
Specialist (*n* = 47)	59.6%	42.6%
Associate Professor (*n* = 15)	66.7%	46.7%
Professor (*n* = 3)	66.7%	66.7%
*p* value (trend)	0.041 (χ² trend)	0.038 (χ² trend)

### Institutional preparedness by specialty

Lipid emulsion availability varied considerably across specialties (Table 6). Anesthesiology departments had the highest reported availability (43.6%), while cardiovascular surgery (25.0%), neurosurgery (23.1%), and obstetrics and gynecology (25.0%) had the lowest. Notably, approximately 30% of respondents across most non-anesthesiology specialties were unaware of the availability status in their unit, reflecting a fundamental institutional communication gap.

**Table 6:** Institutional preparedness (lipid emulsion availability) by specialty

Specialty	Lipid Available (%)	Not Available (%)	Unknown (%)
Anesthesiology	43.6%	33.8%	22.6%
General Surgery	30.8%	42.3%	26.9%
Orthopedics	28.6%	42.9%	28.5%
Obstetrics & Gynecology	25.0%	45.0%	30.0%
Otolaryngology	25.0%	43.8%	31.2%
Neurosurgery	23.1%	46.2%	30.7%
Urology	27.3%	45.5%	27.2%
Cardiovascular Surgery	25.0%	50.0%	25.0%

### Multivariable logistic regression

Multivariable logistic regression identified three independent predictors of correct lipid emulsion dose knowledge (Table 7). Formal LAST training conferred the strongest association (OR 6.38; 95% CI 3.21–12.68; *p* < 0.001), followed by anesthesiology specialty (OR 3.14; 95% CI 1.67–5.91; *p* < 0.001) and senior academic title (OR 2.07; 95% CI 1.08–3.96; *p* = 0.028). Age and gender were not independently associated with correct knowledge (*p* = 0.231 and *p* = 0.785, respectively). The model demonstrated acceptable calibration (Hosmer-Lemeshow *p* = 0.42) and explained 31% of variance in knowledge (Nagelkerke R²=0.31).

**Table 7:** Multivariable logistic regression: predictors of correct lipid emulsion dose knowledge

Variable	OR	95% CI	p value
Formal LAST training (Yes vs. No)	6.38	3.21–12.68	< 0.001
Specialty (Anesthesiology vs. Other)	3.14	1.67–5.91	< 0.001
Academic title (Senior vs. Resident)†	2.07	1.08–3.96	0.028
Age (per year increase)	1.04	0.97–1.12	0.231
Gender (Male vs. Female)	0.92	0.51–1.66	0.785

Dependent variable: correct identification of lipid emulsion bolus dose

*OR*  odds ratio, *CI* confidence interval

†Senior = Specialist, Associate Professor, or Professor. Model Nagelkerke R² = 0.31; Hosmer–Lemeshow p = 0.42

## Discussion

This multi-center cross-sectional study demonstrates substantial and clinically consequential gaps in LAST awareness, knowledge, and institutional preparedness across surgical specialties in Türkiye. The most critical findings were: (1) fewer than one-third of participants correctly identified the lipid emulsion rescue dose; (2) formal LAST training, received by only 41.1% of participants, was the single strongest independent predictor of correct knowledge (OR 6.38); (3) early symptom recognition was absent in more than half of respondents; and (4) fewer than 30% of departments had lipid emulsion immediately available. Notably, even among anesthesiology clinicians, specialists for whom LAST management is a core competency, correct dosing knowledge was present in only 39.0%, raising important questions about the depth and retention of LAST education within anesthesiology training itself. A sub-analysis within the anesthesiology group revealed a marked difference between seniority levels: correct dose knowledge was present in 60.9% of specialists and above (*n* = 23), compared with only 29.6% of anesthesiology residents (*n* = 54). This finding suggests that the overall anesthesiology figure of 39.0% largely reflects the knowledge gap among residents, and that the educational deficit is most pronounced at the early-career stage even within the specialty most responsible for LAST management.

The low rate of correct lipid emulsion dose identification (34.4% overall) is consistent with findings from comparable surveys in other countries and clinical settings. Sagir and Goyal reported similar deficits among postgraduate residents across multiple Indian specialties [12], and Munasinghe et al. documented suboptimal knowledge of LAST management and prevention in a Sri Lankan online survey [11]. Among ophthalmology staff in a standalone eye unit, Subramaniam et al. found that lipid emulsion knowledge and availability were critically lacking [10]. Our data extend these findings to a broader specialty mix and reinforce that insufficient preparedness is a global rather than regionally specific problem [13, 15].

Even among trained participants, the correct response rate was 58.2%—suboptimal for a potentially fatal emergency. This suggests that LAST training, when provided, may not be sufficiently reinforced over time. A single didactic exposure is unlikely to maintain the procedural memory necessary for accurate dosing during a high-stress clinical scenario [16]. The perception of LAST as a theoretical rather than practical competency may also contribute to rapid knowledge decay [6, 14].

The marked inter-specialty disparity, with anesthesiology substantially outperforming all surgical disciplines, was expected, given that LAST is not formally incorporated into most surgical residency curricula or board examination requirements [12, 15, 17]. However, the low absolute awareness rates even within anesthesiology are unexpected and concerning. With anesthesiologists often serving as first responders during perioperative LAST events, this finding suggests that current training structures may not adequately translate knowledge into retained clinical competency.

The significant positive association between seniority and preventive practice adherence (aspiration, ultrasound guidance) likely reflects accumulated clinical experience and the gradual internalization of safety protocols over a career. Nevertheless, the fact that fewer than half of residents routinely performed aspiration before injection indicates that early-career clinicians who administer a large proportion of local anesthetics in training environments represent the group most in need of targeted intervention. It should be noted that aspiration prior to injection, while widely recommended as a precautionary step, is not a reliable method for confirming extravascular needle placement. A negative aspiration does not exclude intravascular injection, as small-gauge needles, bevel positioning, and vessel compression may yield false-negative results [6]. Ultrasound guidance and incremental injection with real-time monitoring therefore remain the more reliable strategies for LAST prevention [6, 8]. The observed low rates of aspiration among residents may thus reflect not only a knowledge gap but also an evolving understanding of its limited predictive value, a nuance that should be incorporated into training curricula. The reasons underlying this low adherence among residents are likely multifactorial. First, inadequate direct supervision during procedural training may result in safety steps being omitted without correction or feedback. Second, time pressure in high-volume training environments may lead residents to prioritize procedural speed over protocol adherence. Third, and perhaps most fundamentally, a systemic failure in safety culture during residency training may normalize the omission of preventive measures when they are not consistently modeled by senior clinicians. Addressing these factors requires not only didactic education but also structured competency-based supervision, with explicit assessment of preventive practice adherence as a formal component of procedural training.

The finding that only 29.2% of participants confirmed immediate lipid emulsion availability in their department is concerning [5, 7, 9]. Approximately one-third of respondents were unaware of its availability status, reflecting a systemic failure of institutional communication and emergency preparedness. Prior studies have associated delays in lipid emulsion access with worse outcomes in simulated LAST scenarios [7, 10, 11]. Mandatory stock requirements and clear labeling in all areas where local anesthetics are administered should be considered minimum institutional standards. It should be noted, however, that these findings derive exclusively from tertiary-level training hospitals; availability and awareness in community or secondary care settings may differ substantially.

Together, these findings suggest four potential areas for institutional improvement:

- Consider implementing simulation-based LAST management training as a regular educational component for all clinicians who administer local anesthetics, irrespective of specialty. Simulation has been shown to significantly improve emergency response accuracy and speed [16]. Future research should employ simulation-based scenarios — encompassing not only LAST but also related perioperative emergencies such as malignant hyperthermia, anaphylactic shock, acute pulmonary embolism, and amniotic fluid embolism — to evaluate how clinicians actually manage these critical events, as theoretical knowledge does not guarantee appropriate emergency response.
- Prioritize the availability and clear labeling of 20% lipid emulsion in all procedural areas, surgical suites, and emergency settings where local anesthetics are used.
- Promote standardization preventive protocols, including aspiration before injection and ultrasound guidance for regional blocks, through institutional guidance, with structured competency assessment for residents.
- Develop and implement systemic point-of-care resources including visual decision aids (dosing charts, LAST wall posters), standardized checklists, and pre-packaged lipid rescue kits in all areas where local anesthetics are administered. These mechanisms are essential to ensure that once LAST is recognized, the clinician is immediately directed to the correct treatment without relying on individual memory recall. The inherent limitations of human memory under rare, high-stress emergencies mean that periodic educational sessions alone are insufficient; systemic preparedness tools must complement individual training to bridge the gap between knowledge acquisition and real-time clinical performance.

At the national level, integration of structured LAST content including dosing thresholds, symptom recognition, and treatment algorithms into surgical residency curricula and board certification requirements would address the systematic knowledge gap identified in this study.

This study was conducted exclusively in tertiary-level training and research hospitals in Türkiye, which represent environments with relatively advanced resources and academic supervision. Community hospitals, district hospitals, and private practice settings where local anesthetics are also widely used were not sampled. It is plausible that awareness and preparedness are lower in those settings, suggesting our findings represent an optimistic upper bound of real-world preparedness in the Turkish healthcare system. International comparisons are constrained by differences in residency training structures, healthcare system organization, and guideline dissemination; however, the convergence of our findings with surveys from Sri Lanka [11], India [12], the United Kingdom [10], and Türkiye [13, 15, 17] suggests broad generalizability of the core conclusions. Nevertheless, the present findings are most directly applicable to the Turkish tertiary hospital context, and caution is warranted in extrapolating to other healthcare settings.

Several methodological limitations inherent to the survey design must be acknowledged. Self-report bias may lead to overestimation of adherence to safety practices (social desirability bias); anonymous participation and voluntary completion were employed to minimize this effect, though their impact cannot be fully eliminated. Online distribution introduced the possibility of participants consulting external resources during completion; however, the high rate of incorrect answers observed suggests this was minimal in practice. Volunteer bias, whereby more knowledgeable participants may be more likely to complete the survey, may have inflated overall awareness rates, implying that real-world preparedness may be even more limited than reported. Participation was restricted to tertiary-level centers, which may overestimate preparedness relative to community or secondary hospitals. Taken together, these biases would generally act in a conservative direction, suggesting that true knowledge gaps are at least as large as those reported here. First, as a cross-sectional survey, causality cannot be inferred. Second, although a post-hoc power analysis confirmed adequate statistical power for primary comparisons, the absence of an a priori sample size calculation and the overrepresentation of anesthesiology (40.1%) represent a potential source of sampling bias that may influence the generalizability of findings across other specialties. The disproportionate representation of anesthesiology participants, a specialty with inherently higher LAST awareness, may have inflated overall knowledge estimates, and specialty-level comparisons for smaller groups should be interpreted with caution. Third, the number of senior academic participants was very small, particularly at the professor level (*n* = 3). Subgroup comparisons and trend analyses according to academic title should therefore be interpreted cautiously, as estimates for senior ranks may be unstable due to limited sample size. Fourth, the instrument used face and content validity but was not subjected to confirmatory factor analysis or criterion validity testing against objective performance measures. Questionnaire-based surveys carry inherent methodological limitations: social desirability bias may lead respondents to report more favorable practices than are actually followed; recall bias may affect the accuracy of self-reported behaviors such as frequency of aspiration or ultrasound use; and perhaps most importantly, there is a well-documented gap between self-reported theoretical knowledge and actual clinical performance under emergency conditions. Participants who correctly identified the lipid emulsion dose on a survey may still fail to recall or apply this knowledge accurately during a real LAST event. Additionally, 18.1% of untrained participants correctly identified the lipid emulsion bolus dose. As the dosing question was open-ended and required participants to write the numerical value without any options provided, correct responses among untrained participants cannot be attributed to educated guessing. This finding most likely reflects knowledge acquired through undergraduate medical education, self-directed study, or incidental clinical exposure rather than formal LAST-specific training. Lastly, the independent effect of formal LAST training on knowledge outcomes may be confounded by specialty background. The majority of formally trained participants were from anesthesiology (52 of 79 trained participants, 65.8%), suggesting that the observed association between training and correct knowledge may partly reflect specialty-level differences in curriculum exposure rather than the effect of training per se. Readers should interpret the training-related odds ratio in the multivariable model with this potential confounding in mind.

## Conclusion

This multi-center cross-sectional study identifies substantial deficiencies in LAST knowledge, preventive practice adherence, and institutional preparedness across surgical specialties in tertiary-level hospitals in Türkiye. Formal training is the strongest modifiable predictor of correct LAST knowledge—yet fewer than half of the surveyed clinicians had received any. The near-universal absence of lipid emulsion in most departments represents an immediately correctable patient safety risk. Targeted simulation-based education, mandatory lipid emulsion availability protocols, and integration of LAST content into surgical training curricula warrant prioritization as patient safety interventions across all clinical environments where local anesthetics are administered. 

## Supplementary Information

Supplementary Material 1.

## Data Availability

The datasets generated and analysed during the current study are not publicly available due to participant confidentiality but are available from the corresponding author on reasonable request.

## Abbreviations

LAST: Local Anesthetic Systemic Toxicity
LA: Local Anesthetic
ASRA: American Society of Regional Anesthesia and Pain Medicine
OR: Odds Ratio
CI: Confidence Interval
SD: Standard Deviation
IQR: Interquartile Range
SPSS: Statistical Package for the Social Sciences
