# Supplementary material for: Awareness and preparedness for local anesthetic systemic toxicity among surgical clinicians: a multi-center cross-sectional survey study
Source: BMC Anesthesiol. 2026 Jul 3;26:575. doi: 10.1186/s12871-026-04073-7 (PMC13602573; doi:10.1186/s12871-026-04073-7)
Supplement: Supplementary file 1 [file 12871_2026_4073_MOESM1_ESM.docx]

# Appendix A. Study Questionnaire (English Translation)

*Original questionnaire was administered in Turkish. The English translation is provided below for reference.*

*Note on question format: The lipid emulsion bolus dose question (Section 5) was open-ended; participants were required to write the dose value themselves. No options were provided. This format eliminates the possibility of correct answers arising from chance, and the observed correct response rate of 34.4% therefore reflects genuine knowledge.*

## Section 1. Participant Information

Age: ___

Gender: (Female / Male)

Department: ___

Years of professional experience: ___

Academic title: Resident	Specialist	Associate Professor	Professor

## Section 2. Training Status

Have you received formal training on local anesthetics and LAST? (Yes / No)

Was the training sufficient? (Yes / No)

Do you have knowledge of local anesthetic dosing? (Yes / No)

## Section 3. Local Anesthetic Use Habits

Types of local anesthetics used (select all that apply):

☐ Lidocaine

☐ Prilocaine

☐ Bupivacaine

☐ Other: ___

Frequency of use:

☐ Every day

☐ Several times a week

☐ Several times a month

☐ Less frequently

Most commonly preferred route of administration:

☐ Subcutaneous

☐ Intravenous

☐ Epidural / Intrathecal

☐ Other: ___

## Section 4. Preparation and Administration of Local Anesthetics

Who typically prepares the local anesthetic?

☐ The physician prepares it themselves.

☐ A nurse prepares it.

☐ A shared process (physician and nurse together).

☐ Other: ___

Who is involved in the preparation of the local anesthetic?

☐ Physician only

☐ Nurse only

☐ Physician and nurse together

☐ Other: ___

Is the prepared local anesthetic verified before administration?

☐ Yes, the physician verifies it.

☐ Yes, the nurse verifies it.

☐ No, it is administered without verification.

☐ Other: ___

Are protocols or guidelines followed during preparation of local anesthetics?

☐ Yes, always.

☐ Usually.

☐ Rarely.

☐ No.

What problems are encountered during preparation? ___

## Section 5. LAST Prevention Methods

Preventive methods used:

☐ Use of correct dosing (Yes / No)

☐ Aspiration before injection (Yes / No)

☐ Ultrasound-guided administration (Yes / No)

☐ Other: ___

Is 20% lipid emulsion available in the hospital? (Yes / No / I don't know)

If yes, do you know where it is stored? (Yes / No)

**Knowledge of LAST Management (open-ended — participants write the value themselves):**

Bolus dose: ___ mL/kg	(I know / I don't know)

Maintenance dose: ___	(I know / I don't know)

## Section 6. LAST Symptoms

Knowledge of early symptoms (Yes / No):

1. ___

2. ___

3. ___

Knowledge of late symptoms (Yes / No):

1. ___

2. ___

3. ___

## Section 7. Management and Follow-up

Have you encountered a LAST event? (Yes / No)

Do you know the recommended monitoring duration after LAST?

After seizure: ___

After cardiovascular instability: ___

## Section 8. Additional Notes

Investigator observations: ___

Additional information / comments: ___
